# Enhancing shared and surrogate decision making for people living with dementia: A systematic review of the effectiveness of interventions

**DOI:** 10.1111/hex.13167

**Published:** 2020-11-28

**Authors:** Andrew Geddis‐Regan, Linda Errington, Clare Abley, Rebecca Wassall, Catherine Exley, Richard Thomson

**Affiliations:** ^1^ School of Dental Sciences Newcastle University Newcastle upon Tyne UK; ^2^ Population Health Sciences Institute Newcastle University Newcastle upon Tyne UK; ^3^ Newcastle upon Tyne Hospitals NHS Foundation Trust Newcastle upon Tyne UK; ^4^ Faculty of Medical Sciences Newcastle University Newcastle upon Tyne UK

**Keywords:** cognitive impairment, decision making, dementia, proxy decision making, shared decision making

## Abstract

**Background:**

Dementia can have a profound impact on decision making. People living with dementia (PLwD) often need to make decisions about health care, and, as dementia progresses, decisions may need to be made on their behalf. Specific interventions may support this process.

**Review Question:**

What interventions are effective in improving shared decision making or surrogate decision making on the health care of PLwD?

**Methods:**

A narrative systematic review of existing literature was conducted. Seven databases, grey literature and key journals were searched. After exclusion by title, abstracts then full texts were reviewed collaboratively to manage any disagreements.

**Results:**

Eight studies met the inclusion criteria. Two articles, including one RCT, evaluated decision aids regarding the use of enteral feeding in advanced dementia. Six further articles, including five RCTs, were found which evaluated the effectiveness of interventions supporting patients or carers with advance care planning.

**Conclusion:**

Decision‐making interventions typically consist of multiple components which aim to establish preferences for future health care. Advance care planning interventions supported aspects of the decision‐making processes but their impact on decision quality was rarely evaluated. Interventions did not increase the concordance of decisions with a person's values. The decision‐specific interventions are unlikely to produce benefit in other decision contexts.

**Patient Involvement:**

Two caregivers, a public stakeholder group and a carer group were consulted in the design of the wider study to which this review relates. Six PLwD refined the research questions addressed in this paper.

## 1 INTRODUCTION

Dementia is a term encompassing multiple neurodegenerative conditions, including Alzheimer's disease and vascular dementia.^1^ Dementia is highly prevalent, with estimates predicting the condition will affect 75 million people worldwide by 2030.^2^ Different types of dementia arise from different pathophysiological processes but have some common symptoms These can include impaired memory and functional difficulties both of which can affect a person's ability to independently perform activities of daily living.^1^ Decisions about health care are frequently necessary for people living with dementia (PLwD) both regarding dementia care itself and in relation to any co‐existing conditions. Examples of more complex decisions include those related to end‐of‐life care,^3^ moving into residential care^4^ or dental care.^5^

Shared decision making (SDM) is a model for clinical practice which incorporates patients’ values and preferences and supports them to make decisions about their own care in collaboration with clinicians.^6^, ^7^, ^8^ This approach has been promoted in policy^9^, ^10^, ^11^ as it emphasizes a patient's autonomy^6^, ^7^ through information sharing in both directions between patient and clinician.^12^, ^13^ There is a broad spectrum of perspectives on what constitutes SDM for PLwD.^14^ As dementia progresses, communication and decision making may require more support from family members or carers yet studies have identified a dissonance between person's preferences and the perceptions of their caregivers.^15^ There is a need for their knowledge of a person to be considered holistically alongside other factors relevant to decision making.

Where dementia progresses a specific decision may arise where decisional capacity is determined to be lacking. In this situation, decisions are made by a *surrogate* or *substitute* decision maker, typically a family member. The point at which decisional capacity is lost; the person who makes a decision; and the bioethical approach used to inform a decision varies based on international legislation.^16^ Substituted Judgement is commonly used to refer to the trying to take the course of action a person would have chosen themselves.^17^ This approach considers a person's values and preferences but fails to identify how they might feel in light of any new or worsening cognitive impairment.^18^ The *best interests* standard is an alternative approach and is the cornerstone of the Mental Capacity Act (MCA) for England and Wales.^19^ When making a decision in somebody's best interests, a holistic view of a person's welfare alongside their expressed values and preferences should be used to inform any decisions made for their care.^20^ The Mental Capacity Act does not define *best interests* explicitly^21^ though much has been written about both its scope and its perceived ethical superiority over *substituted judgement*.^18^, ^20^

Legal roles specified in the MCA, such as Lasting Power of Attorney or a Deputyship, allow named individuals to act as a decision makers.^21^ In the absence of formal roles, a family member may support clinical decision making by providing insight into the PLwD’s values and preferences.^19^ The potential for the views of the PLwD, family and clinicians to differ can complicate best interests decision‐making process. PLwD can exercise their autonomy by making their wishes known in an Advance Care Plan (ACP) before any loss of decisional capacity. An existing review, however, has found that ACPs are only limited in their effectiveness for PLwD.^22^

Decision aids (DAs) are one approach commonly described in the literature yet other approaches can also support SDM.^23^ DAs are tools that support patients or surrogate decision makers by providing information and supporting them to make the decision that is right for them .^24^ DAs have been reviewed systematically both in general^24^ and in relation to PLwD.^25^, ^26^ However, the effectiveness of DAs or other interventions are not comprehensively understood in supporting shared or surrogate decision making with or for PLwD. It could be suggested specific approaches to optimize communication, modify expectations or support value elicitation could all potentially enhance decision making for PLwD separately or alongside DAs. This review aims to explore the effectiveness of interventions that aim to support shared decision making with or for PLwD facing health‐care decisions. Doing so should help inform the development of future DAs or other interventions to support this patient group and those involved in their care.

### 1.1 Review question

What interventions are effective in improving shared decision making or surrogate decision making in relation to the health care of PLwD?

## 2 METHODS

### 2.1 Design

A systematic review was undertaken to identify studies that assessed the effectiveness of interventions relevant to health‐care decision making with or for PLwD. Working with PLwD and carers helped to focus the research question and the need to know *‘what actually works in the real world’*. It was anticipated that studies of effectiveness would typically be quantitative yet qualitative studies were not explicitly excluded as these may shed light on the processes of implementation and the factors contributing to effectiveness. The review aligns with guidance from PRISMA Preferred Reporting Items for Systematic Reviews and Meta‐Analysis.^27^ The protocol was prospectively registered on PROSPERO (CRD42019154707), accessible at: https://www.crd.york.ac.uk/PROSPERO/display_record.php?RecordID=154707.

### 2.2 Inclusion and exclusion criteria

The inclusion and exclusion criteria are shown in Table 1. Systematic reviews were excluded but were read to identify other relevant studies. There were no limitations on date or geographic region.

**Table 1:** Inclusion and Exclusion criteria and their justifications

	Justification for criteria
Inclusion Criteria	
1) The paper assesses effectiveness of an intervention aiming to support shared or surrogate decision making for PLwD	The review is aiming to determine the effectiveness of interventions, not simply to describe a list of untested interventions.
2) The paper examines decisions about health‐care interventions	For non‐health‐care decisions, such as those relating to finance or social care, a range of different factors could be identified that do not translate to health‐care contexts.
3) The decisions studied are *actual* decisions made as opposed to hypothetical decisions	The effectiveness of an intervention cannot be definitely established by testing it against hypothetical or simulated decisions.
Exclusion Criteria	
1) Studies of interventions that had not been evaluated	These are unable to identify effectiveness
2) Studies exploring decisions related to cardio‐pulmonary resuscitation (CPR) alone	The decision to refuse CPR is unique and highly emotive, relating to refusing that may sustain life or allow death. The unique nature of this refusal decision means literature exploring this specific decision in an end‐of‐life context would not support other health‐care decisions.^28^
3) Studies related to non‐health‐care decisions	See above.
4) Studies not specifically considering dementia,	Interventions supporting decisions outside of the context of dementia are not the focus of this review.
5) Studies exploring only the clinician's role in decision making	Patients or surrogate decision makers should be involved in decision making. Where this is not the case there is little to be learned about enhancing decision making for PLwD themselves
6) Studies in languages other than English.	There were insufficient resources to facilitate translation.

### 2.3 Outcomes of interest

Fixed outcomes of interest were not specified due to the wide range of measures used to evaluate shared or surrogate decision making and the lack of standardization in assessment.^29^ The process by which a decision is made and the quality of a decision itself can be evaluated by multiple measures.^29^, ^30^ These include assessments of satisfaction, value concordance and patient involvement such as by the OPTION scale.^31^, ^32^ It was anticipated that the effectiveness of interventions would be evaluated using any of these measures, additional measures of communication or a variety of combinations, thus preventing formally specified fixed outcomes of interest.

### 2.4 Search strategy

Seven databases were searched with a strategy using MeSH headings and keywords with support of a subject‐specific librarian (LE). The strategy was informed by a P‐I‐C‐O design in which Population: PLwD/carers, Intervention: any intervention aiming to improve decision making, Comparator: usual standard of care (for controlled studies) and Outcome: improvement in decision making. The strategy used in Medline via OVID is shown in Appendix 1. Search strategies were intentionally broad due to the lack of a single intervention of interest and the identification, via the pilot search, that the term ‘intervention’ is inconsistently used in MeSH headings, keywords or titles of relevant studies. The search strategy was modified for each separate database. The following databases were searched on 16 December 2019: Medline, Embase and PsycINFO (via OVID), CINAHL via EBSCO, SCOPUS, Web of Science and the Cochrane Library. Grey literature was searched via OpenGrey using keywords. Specific hand searches were undertaken of key journals, namely *Medical Decision Making, Health Expectations* and *Patient Education and Counseling* dating to their first issues. Reference lists of identified relevant studies and systematic reviews were also reviewed to identify relevant studies. The search was re‐run on 10 July 2020. No further relevant studies were identified.

### 2.5 Study selection

**Figure 1:**
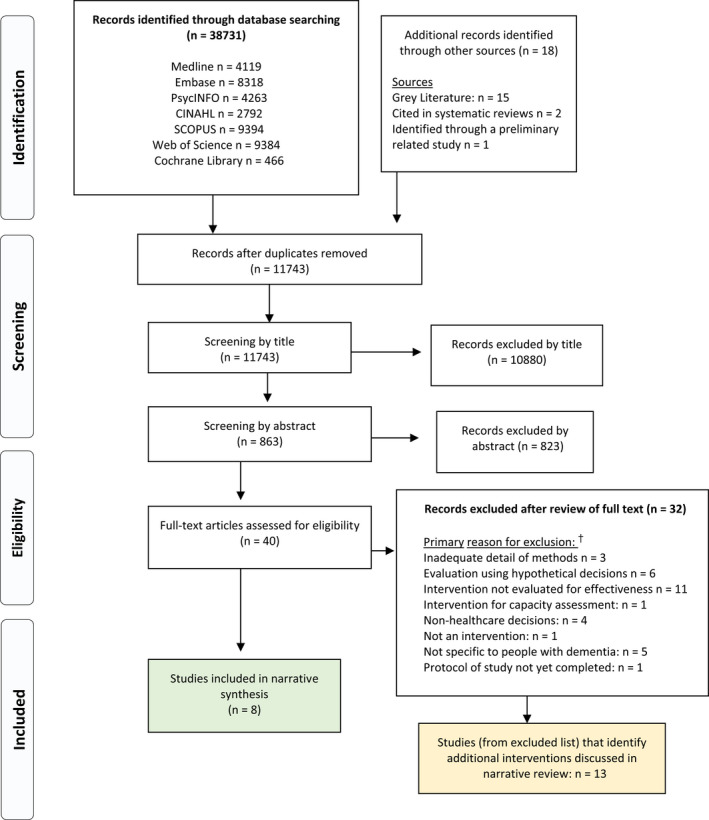
PRISMA Flow diagram. ^†^Multiple studies were excluded for more than one of the below reasons: the most influential factor leading to exclusion is detailed here

After removal of duplicates, study titles were screened against inclusion and exclusion criteria by one author (AGR). Studies were included for review at the subsequent stage when there was any uncertainty about inclusion. For the remaining studies, two authors (AGR and LE) reviewed all abstracts using Rayyan.^33^ After screening the first 100 abstracts, the interpretation of inclusion and exclusion criteria was reviewed to establish agreement in study inclusion. Any uncertainty or disagreement about inclusion was discussed in‐depth and, where necessary, resolved by discussion with a third reviewer (RT). Full texts were reviewed by AGR, and any uncertainty about inclusion was discussed with RT. Pilot or feasibility analyses identified were read to assess whether or not the study aimed to assess effectiveness formally. Figure 1 shows the PRISMA flow diagram.

### 2.6 Data extraction and quality assessment

The outcomes specified in the protocol were extracted onto a specific data‐extraction spreadsheet which was used to populate Table 2. The McGill Mixed Methods Appraisal Tool (MMAT)^34^ was used for quality assessment. Studies were checked against the MMAT quality criteria corresponding with the study type. Studies were not excluded based on poor quality. The identification of methodological limitations informed the narrative discussion related to the studies included.

**Table 2:** Summary of included studies

Study Authors Country Study Design	Aim of study	Nature of Intervention	Participants	Outcome Measures	Key findings
Studies of Interventions to aid feeding decisions					
Hanson *et al* (2011)^38^ USA Cluster RCT	To test whether a decision aid improves quality of shared decision making about feeding options in advanced dementia.	A printed decision aid modified from that presented in Mitchell *et al* 2001.^45^ Modifications included a revised reading level and simplified sentences with increased font size.	256 dyads of people with late‐stage dementia and their family/member or power of attorney (127 dyads in intervention, 129 dyads in control)	Knowledge Expectation of benefit, Decisional Conflict at 3 months, Frequency of Discussions about options.	The intervention improved knowledge scores and lowered decisional conflict after 3 months. Surrogates more often discussed feeding options with a health‐care provider. Specific results in Table 3
Snyder *et al* (2013) ^37^ USA Controlled Before‐after test of intervention with qualitative enquiry	To describe surrogates’ baseline perceptions of feeding options in dementia and to determine if a decision aid alters surrogate decision makers’ knowledge, expectation of benefit from tube feeding, decisional conflict, and preferred feeding approach	As above: Hanson *et al* (2011)^38^	255 surrogate decision makers of nursing home residents with advanced dementia and feeding problems (126 intervention, 129 control).	Knowledge, decisional conflict, expectations of benefit, preferred approach and certainty of preferences.	Intervention surrogates had improved knowledge scores, decreased expectation of benefits from tube feeding and reduced decisional conflict. Specific results in Table 3
Studies of Advanced Care Planning Interventions					
Ampe *et al* (2017)^39^ Belgium Before and After Study with control group	The first aim was to evaluate the influence of ‘we DECide’ on the policy and practice of ACP in nursing home dementia care units. To investigate the staff's perceived barriers and facilitators for implementing SDM in ACP.	WeDECide: An educational intervention to support nursing home staff to use shared decision making in relation to conversations about advanced care planning for PLwD	90 nursing home staff members in a range of roles from 18 different dementia care units. 21 conversations (11 in intervention group, 10 in control group) were recorded (with residents and family members) for analysis	Degree to which ACP was discussed Degree of involvement of residents and families using OPTION‐12. Perceived barriers and facilitators.	‘We DECide’ had a positive influence on advance care planning policy. Daily practice, however, did not change: ACP was not discussed more frequently after the intervention, nor between the intervention and control group The degree of involvement of residents and families in SDM was no different before or after the intervention.
Brazil *et al (*2018)^42^ UK Paired Cluster RCT	To evaluate the effectiveness of advance care planning with family carers in dementia care	A combination of components: a trained ACP facilitator, family education, family meetings, documentation of advance care plan decisions, and orientation of GPs and nursing home staff to the intervention.	142 family members of residents with dementia (51 in intervention group, 91 in control group) for main analysis	Family carer uncertainty in decision making via Decisional Conflict	ACP reduced carer uncertainty in decision making. There was evidence of a reduction in total Decisional Conflict Scale score in the intervention group compared with the usual care group (−10.5, 95% confidence interval: −16.4 to − 4.7; *P* < .001).
Goossens *et al* (2019)^43^ Belgium Cluster RCT	To assess the effects of the intervention on (1) the level of SDM in ACP for persons with dementia in nursing homes, (2) the perceived importance, competence and frequency of staff members concerning SDM and (3) the facilitating and hindering context elements for the sustainability of the training result	WeDECide optimized: a complex intervention consisting of 2 workshops designed to assist patients specifically in making choices about health care through following the ‘steps’ of SDM (choice talk, option talk, decision talk). A development of the intervention detailed by Ampe *et al,* 2017^39^	311 staff members from 65 nursing home wards: 316 audio recordings included staff, patients and a family member	Level of SDM during formal ACP conversations between residents, families and staff members. Perceived competence in SDM, perceived importance of SDM and frequency of use of SDM.	The level of SDM increased in the intervention group (*P* < .001), which persisted at 6 months. Time spent on discussions did not increase. Staff participants felt more competent in SDM (*P* = .01) and felt it was more important (*P* = .031) though it was not used more frequently (*P* = .201)
Hanson *et al* (2017)^40^ USA Single‐blind cluster RCT	To test if the Goals of Care (GOC) decision aid improves the quality of communication and palliative care for nursing home residents with advanced dementia	A 'Goals of Care' Video Decision aid: 18‐minute Goals of Care video decision aid and a structured meeting between the surrogate and the interdisciplinary care plan team at the nursing home after 3 months	302 dyads: people with advanced dementia and their family decision makers. (151 intervention, 151 in control group)	Quality of communication scores, concordance with clinicians on goals of care and degree of concordance of treatment with expressed preferences	The intervention group showed better quality of communication (6.0 vs 5.6 *P* = .05 at 3 months) and higher ratings of end‐of‐life communication (3.7 vs 3.0, *P* = .02).Goal concordance did not differ at 3 months. At 9 months or death, decision makers in the intervention group perceived greater concordance with providers compared with the control group (88.4% vs 71.2%, *P* = .0001)
Hanson *et al* (2019)^41^ USA Pilot RCT	To test dementia‐specific specialty palliative care triggered by hospitalization	Protocolized specialty palliative care consultation whilst hospitalized, plus 2‐week post‐discharge transitional phone support by a palliative care nurse practitioner.	62 dyads of people living with late‐stage dementia and family decision makers (32 intervention, 30 control).	Secondary Decision‐related outcomes: Discussion of prognosis, goals of care, completion of Medical Orders for Scope of Treatment and treatment decisions.	Intervention families were more likely to discuss prognosis (90% vs 3%, *P* < .001) and goals of care (90% vs 25%, *P* < .001), and to have a Medical Order for Scope of Treatment at 60‐day follow‐up (79% vs 30%, *P* < .001).
Whitlatch *et al* (2019)^44^ USA RCT	To evaluate the effectiveness of the SHARE program, considering the immediate effects of SHARE on each member of the patient‐carer dyad.	SHARE (Support, Health, Activities, Resources and Education): A psycho‐educational programme using counselling approaches to support people living with dementia and their family caregivers to consider future care preferences	128 patient/carer dyads (totalling 256 participants): 44 dyads in in control group, 84 dyads in intervention group)	Care preferences, service use, dyadic functioning, emotional disruption, positive and negative affect. Satisfaction.	Dyads in the intervention group were able to construct care plans and had higher use of services. Of five areas related to satisfaction, there was increase only in relation to the counsellor for PLwD (*P* < .01). The caregivers reported satisfaction with four of the five domains of satisfaction assessed (*P* < .05). In the intervention group dyadic functioning improved in only one dimension: decreased emotional disruption. There was no increase in care‐related agreement, in positive interactions or in negative interactions.

### 2.7 Analysis and synthesis processes

The heterogeneity of studies and measures used meant a meta‐analysis was not possible. A narrative review was employed to broadly reflect a thematic summary approach^35^ whereby studies are categorized into groups and discussed based on their key features. This approach allowed a detailed narrative exploration and a comparison of studies presenting interventions with similar aims.^36^

## 3 RESULTS

### 3.1 Study characteristics

Eight studies met the criteria for inclusion (Table 2). Two studies assessed interventions related to enteral feeding in advanced dementia, a controlled before and after study^37^ and a randomized trial.^38^ Six studies evaluated interventions related to ACP with either caregivers alone or PLwD‐caregivers dyads; these included one controlled before and after study^39^ and five randomized trials.^40^, ^41^, ^42^, ^43^, ^44^ All were undertaken in either the USA, the UK or Belgium. DAs featured in both studies regarding enteral feeding decisions^37^, ^38^ but only in one study related to ACP.^40^ The remaining studies detailed interventions aiming to support multiple decisions as opposed to tackling one specific issue.

The sharp divide between the type of decision being made in the included studies warranted a separate discussion for each category. This differs from the protocol which intended to separate studies by decisions made by or for the PLwD. The heavy emphasis on surrogate decision making and future care planning made it necessary to redefine how the studies would be separately examined.

### 3.2 Overview of DAs for feeding decisions

Decisions aids for enteral feeding decisions were targeted at surrogate decision makers in advanced dementia. Both studies^37^, ^38^ used a modified version of the DA initially described by Mitchell *et al* ^45^ The studies were led by the same researcher with the later study^37^ describing itself as undertaking ‘baseline interviews’ as part of a larger trial, citing the RCT.^38^ Though one study precedes the other, it was published at a later time and both provide separate insights into the effectiveness of the intervention in different contexts.

### 3.3 Quality assessment of studies of DAs for enteral feeding decisions

Reviewed against the MMAT, the RCT^38^ has the potential for bias as the method of randomization is unclear and incomplete outcome data are presented. The use of cluster randomization was necessary, and a control was employed. Assessors were not blinded potentially leading to bias. The before and after study^37^ had complete follow‐up, though the methods were not comprehensively described in either quantitative or qualitative components. This limits the understanding of the specific approaches used. Neither study provided a power calculation yet sample sizes were similar. The distribution of participants across intervention and control groups was also comparable.

### 3.4 Summary of included studies of DAs for enteral feeding decisions

Snyder *et al,* ^37^ interviewed surrogate decision makers before and after exposure to the DA and used a list of questions to gather decision makers’ perceptions. The studies explored similar outcome measures as shown in Table 3, specifically knowledge, decisional conflict and expectation of benefit. Hanson *et al*,^38^ also explored the impact of an intervention on the frequency of relevant discussions between family members and clinical staff. Across the studies, a total of 511 surrogate decision makers were included. Snyder *et al* ^37^ describe that, after exposure to the DA, participants became more aware of the limitations and challenges related to artificial feeding. However, this concept arose from the interviews undertaken and the authors note this is not a demonstration of effectiveness. Measured quantitatively, surrogates were significantly less likely to expect artificial feeding to provide benefits to the PLwD. Table 3 shows the impact of the interventions on the outcome measures common to both studies. Decisional conflict was reduced and knowledge increased; though both changes were statistically significant the real‐world extent of changes was minimal. Though the studies demonstrate effectiveness, the magnitude and impact of this effectiveness was limited.

**Table 3:** A summary of key outcomes from studies evaluating decision aids related to enteral feeding

Study	Outcome					
	Surrogate Decisional Conflict^a^		Surrogate Knowledge (%)^b^		Expectation of Benefit^c^	
	Pre	Post	Pre	Post	Pre	Post
Hanson *et al* (2011)^38^		Intervention: 1.65 Control: 1.97*		Intervention: 88.4 Control: 79.4*		Intervention: 2.3 Control: 2.6*
Snyder *et al* (2013)^37^	2.24	1.91**	81.6	88.4**	2.73	2.32**

^a^ Using Decisional Conflict Scale.

^b^ Converted from scores to percentages.

^c^ Using Expectation of Benefit Scale (scored 1‐4).

* Mean (standard deviation) <0.01,

** Mean (standard deviation) <0.001.

### 3.5 Overview of ACP interventions

Only one study focused specifically on participants living with dementia at an early stage, where they were paired with family members in dyads.^44^ Other studies included people living with later stages of dementia, also as dyads,^40^, ^41^ and one study included only caregivers as participants for those with late‐stage dementia.^42^ The remaining two studies^39^, ^43^ detail approaches that could be employed with PLwD, caregivers or both as the course of dementia progressed. A range of evaluation measures were used, including decisional conflict,^42^ frequency of relevant discussions^39^, ^43^ the quality of communication,^40^ levels of SDM,^39^, ^43^ the number of palliative care domains discussed,^40^, ^41^, ^42^ family report of concordance with clinicians on the primary goal of care,^40^ decision satisfaction^40^, ^42^, ^44^ and dyadic functioning.^44^ The variation in outcome measures reflects the variation in study design, the primary outcome measure chosen and the specific features of each intervention.

### 3.6 Quality assessment of studies of ACP interventions

The assessment of the five RCTs against the MMAT is shown in Table 4. The study presented by Ampe *et al,* ^39^ mirrors the RCT from Goossens *et al,* ^43^ in that they both only consider nursing home staff as participants and not PLwD. Both studies used blinded assessors but were unclear in describing the method of randomization. The remaining studies related to ACP are relatively consistent in their quality (Table 4). These studies consistently used appropriate randomization and recruited comparable baseline groups. Complete outcome data are rarely presented yet this is often a feature of studies involving participants nearing the end of life. The lack of blinding relates to cluster randomization where assessors were aware of the groups to which participants had been assigned. Specific features of studies impacting on their quality are detailed in the description of each study below.

**Table 4:** Assessment of randomized studies against MMAT criteria

	Brazil *et al* (2018)^42^	Goossens *et al* (2019)^43^	Hanson *et al* (2017)^40^	Hanson *et al* (2019)^41^	Whitlatch *et al* (2019)^44^
Is randomization appropriately performed?	Y	U	Y	Y	Y
Are the groups comparable at baseline?	Y	Y	Y	Y	Y
Are there complete outcome data?	N	N	Y	N	N
Are outcome assessors blinded to the intervention provided?	Y	Y	Y	N	N
Did the participants adhere to the assigned intervention?	Y	N	Y	Y	Y

Note

Criteria taken from Mixed Methods Appraisal Tool.^34^

N, no; U, unclear; Y, yes.

### 3.7 Summaries of studies of ACP interventions

Whitlatch *et al,* ^44^ assessed the effectiveness of the SHARE program which actively involved working with the PLwD and carers to address the dyad's concerns about future care. The programme aimed to support person‐centred care delivery. Compared with the control group, who received a single episode of professional support, an increase in satisfaction across multiple measures was observed for caregivers only. The PLwD reported increased satisfaction only related to the counsellor who supported the intervention or control (*P* < .01). There was no significant difference in the care‐related agreement between PLwD and caregivers or in the functioning of the dyadic relationship. The intervention appears effective only in supporting service use and construction of ACPs. It does not appear to affect the extent to which a person's values and preferences are considered in the process of producing ACPs.

The work of Ampe *et al* ^39^ is built upon by Goossens *et al* ^43^ where the *WeDECide* and *WeDECide Optimized* practitioner training interventions are described, respectively. The ‘optimized’ version^43^ aimed to actively emphasize the importance of involving PLwD and family members in communication. These studies use audio‐recorded ACP consultations to examine the process of SDM. The participants are described as nursing home staff, not PLwD or family members. Whilst Goossens *et al,* ^43^ present a sample (n = 311) that aligned with the number of recordings analysed (n = 316), the earlier study^39^ included 90 participants but only analysed 21 consultations. No justification or explanation is given for why so few consultations were included. Regardless of this limitation, Ampe *et al,* ^39^ demonstrated the intervention improved the extent to which policy in the units involved in the study complied with best practice when assessed by the ACP‐audit tool. There was no change in the extent that SDM was used in ACP conversations. In contrast, the RCT not only demonstrated an increased level of SDM (OPTION‐12 Score 24.98 vs 53.49, *P* < .001) but demonstrated that this persisted at the six‐month follow‐up (OPTION‐12 Score 21.27 vs 56.00, *P* < .001) having controlled for participant characteristics and cluster effects.^43^ Despite this finding, there was no associated increase in the frequency with which the nurses said they used SDM nor in the time spent in ACP conversations.

The *Goals of Care* intervention^40^ involved a video DA followed by a meeting between the surrogate decision maker and the care team three months after its use. Its effectiveness was explored in a single‐blind randomized trial with 302 PLwD‐family member dyads. Outcome measures were examined at baseline then at three, six and nine months or death. Those exposed to the DA demonstrated a modest improvement in communication quality at three months (Quality of communication: 6.0 vs 5.6 *P* = .05) though an increase in family report of concordance with physicians on goals of care only became apparent by nine months or death.

Brazil *et al*,^42^ studied an intervention where an ACP educator met with families and discussed their preferences. Decisional conflict was the primary outcome measure and was reduced in the intervention group compared to those receiving usual care (reduction of 10.5, *P* < .001). Considering future care preferences with the support of an ACP educator meant that the surrogate decision maker was less conflicted and more certain about the decisions expressed in the ACP.

Hanson et al^41^ evaluated a palliative care approach used following a hospital admission including post‐discharge support. The number of post‐discharge hospital visits was the primary outcome measure whilst decision making was only assessed in secondary outcomes. Those in the intervention group were more likely to avoid hospitalization and receive hospice care. Within the decision‐related outcomes, the intervention group showed an increase in frequency of discussions of prognosis (90% vs 3%, *P* < .001) and discussions about goals of care (90% vs 25%, *P* < .001). The study concluded that the intervention improved decision making for PLwD approaching the end of life; this can be attributed to the improvement in the process of decision making that the intervention produces.

### 3.8 Interventions Identified that failed to fulfil inclusion criteria

Within the 32 papers excluded at full‐text stage, a further 10 interventions related to decision making for PLwD were described across 13 publications. These are summarized in Table 5. Reasons for exclusion from the primary review included hypothetical decisions,^46^, ^47^, ^48^, ^49^ studies not exploring effectiveness,^50^, ^51^, ^52^, ^53^, ^54^, ^55^, ^56^ a focus on non‐health‐care decisions^57^ or outcomes unrelated to decision making.^58^ Despite reasons for exclusion, there is merit in a narrative exploration of these interventions as many relate to large‐scale funded trials or have undergone earlier testing which may precede studies of effectiveness.

**Table 5:** Additional interventions identified in excluded studies

Study	Intervention Name	Description of Intervention
Bonner *et al* (2014)^56^	Advance Care Treatment Plan (ACT‐Plan) for African American Family Caregivers	A group‐based education intervention, with dementia caregivers where a number of end‐of‐life discussions were had to inform future decisions from the patient's perspective.
Dassel *et al* (2019)^55^	The LEAD Guide (Life‐Planning in Early Alzheimer's and Dementia)	A dementia‐focused instrument that can be used by those with early dementia, family members and clinicians to document the persons with dementia's preferences and values to inform current or future care.
Górska *et al* (2016)^54^	Family Group Conferencing	A five‐stage process in which service users, family members, and health/social care professionals come together into a family‐led decision‐making forum.
Loizeau *et al* (2019)^49^	Fact Boxes	Fact Boxes: short paper‐based tools to support a variety of decision makers, presenting the benefits and harms of treatment approaches using simple language. The examples studied are artificial hydration and antibiotic therapy in pneumonia in PLwD.
Murphy and Oliver (2012),^53^ Reitz and Dalemans (2016)^57^	Talking Mats	Talking Mats uses a simple system of picture symbols, placed on a textured mat, that allow people to indicate their feelings about various options within a topic by placing the relevant image below a visual scale.
Reinhardt *et al* (2014)^58^	A proactive discussion with carers about end‐of‐life care	A face‐to‐face, structured conversation about end‐of‐life care options with family members of nursing home residents with advanced dementia.
Saini *et al* (2016)^51^	Compassion intervention	An intervention delivered by an interdisciplinary care leader, in relation to people with advanced dementia nearing end of life aiming to promote integrated care, to educate staff, to support holistic assessments and discuss end of life with families.
Sampson *et al* (2011)^50^	Palliative assessment	A palliative care patient assessment which informed an ACP discussion with the carer, who was offered the opportunity to write an ACP for the person with dementia.
Song *et al* (2019)^52^	SPIRIT (Sharing Patient's Illness Representation to Increase Trust)	SPIRIT is a counselling intervention conducted with the patient with early dementia and surrogate together to promote authentic dialogue between them (not just to complete an advance directive document).
Volandes *et al* (2009a),^46^ (2009b),^47^ (2011)^48^	Video Decision Aid	A 2‐minute video decision support tool visually depicting a patient with advanced dementia to identify and specify preferences if dementia were to develop.

Only one DA was identified in the excluded studies; Volandes *et al*,^46^, ^47^, ^48^ detail a video decision support tool used with older participants who may develop dementia. Those in the intervention groups exhibited a reduction in decisional conflict and an increase in knowledge. The video DA was evaluated in relation to a hypothetical decision with people who were not living with dementia which limits any understanding of how effective it may actually be in its intended context.

Several interventions focus on the PLwD at the earlier stage of dementia.^54^, ^55^, ^57^ Talking Mats is presented as a practical approach to support PLwD to communicate their preferences^53^, ^57^; this has not been evaluated for effectiveness but could potentially enhance communication and support participation in SDM. Other interventions target either a patient‐caregiver dyad or the family member alone to establish preferences to inform future care.^52^, ^54^, ^55^, ^56^ Górska *et al,* ^54^ present a family conferencing schedule that can be used to support care for a PLwD at any stage. This study identified a generally positive perspective of the Family Group Conference approach but could not demonstrate effectiveness via qualitative methods alone.

The SPIRIT study, discussed by Song *et al* ^52^; examines feasibility as opposed to effectiveness yet suggests a counselling intervention can support PLwD to articulate their wishes. The Compassion Intervention detailed by Saini *et al,* ^51^ also aimed to bring multiple parties together using ‘Interdisciplinary Care Leaders’ to support end‐of‐life discussions. A qualitative evaluation was used with the intervention in place. This suggested that using an individual practitioner with appropriate resources could improve decision‐making practice related to end‐of‐life care in dementia.

Other studies have examined separate approaches to support surrogate decision makers for PLwD.^49^, ^50^, ^51^, ^58^ One example is ‘Fact Boxes’^49^ which present factual information to support decision making for specific decisions. These were evaluated using hypothetical scenarios meaning their effectiveness in in real decision‐making contexts cannot be established.^49^ Paralleling the included study from Hanson *et al* ^41^ the study from Sampson *et al,* ^50^ aimed to assess an intervention designed to support carers to produce ACP following unplanned hospital admissions for PLwD. In this feasibility study, decisional conflict decreased in the intervention group though the extent and statistical significance of this is not clear. This study suggests that specific palliative care assessments and support did not increase the frequency of ACP production. Similar approaches are employed by Reinhardt *et al*,^58^ whose intervention contained structured conversations with palliative care teams. This randomized trial considered satisfaction with care as its primary outcome without emphasis on any decision‐related outcomes.

### 3.9 Components of interventions

Within the interventions in the included studies (Table 2) and those excluded (Table 5) the most common components appear to be tools to improve communication between a range of parties such as patients, family and health‐care professionals. A separate common ingredient is the documentation of future preferences (such as in a formal ACP) by either patients or a surrogate decision makers. Interventions to provide decisions makers with information are also common, with written information appearing to be frequently discussed. Education is also commonly featured as a component of interventions either for health‐care professionals, residential care staff or for patients or their caregivers.

## 4 DISCUSSION

Despite the differing focusses of the included studies, the randomized trials related to ACP all demonstrated some benefit to the overall decision‐making process even if this was not apparent across all outcome measures. Though negative findings appeared in some domains, this review found that changes in clinical practice can effectively improve aspects of decision making for PLwD or family carers. Communication is frequently considered either directly or in relation to decision making, and it is unsurprising that improved communication leads to greater satisfaction with decision‐making processes.

### 4.1 The role of DAs

DAs were effective in relation to supporting decisions about the use enteral feeding in advanced dementia. For ACP, the only study that evaluated a DA^40^ demonstrated an impact on goal concordance only after a nine‐month follow‐up. Assessment of goal concordance considered the goals of the family member and clinician, not the goals of the PLwD. Whilst the DA may have been effective, the outcome measures used mean that only limited insight into its effectiveness can be gained. This insight becomes almost dismissible when the views of the PLwD themselves are considered in the wider assessment of effectiveness. A Cochrane review has demonstrated how DAs can improve knowledge and clarify the decision makers’ values.^24^ When capacity is retained or only minimally impaired, the use of existing decision‐specific DAs may be sufficient to support PLwD at an earlier stage and specific DAs may be unnecessary.

Two separate systematic reviews have specifically examined decision aids related to decisions with or for PLwD.^25^, ^26^ These reviews explore DAs in general and include reviews that did not specifically focus on effectiveness. Both papers highlight key issues which are reflected in the studies included in this review. Crucially, only single decisions are included in the DAs identified, despite many PLwD facing multiple complex health‐related decisions.^25^ Though this is an inherent feature of DAs, it raises the question of how appropriate this approach is for people with multiple longer‐term conditions alongside cognitive impairment. In addition, the design of DAs and the focus on surrogate decision makers becomes clear when existing reviews are revisited.

### 4.2 The suitability of outcome measures

Many outcome measures used, particularly measures of decisional conflict and communication relate only to the *process* of decision making. Outcome measures used, especially related to ACPs, may have limited the ability of included studies to demonstrate the effectiveness of interventions described. A challenge in identifying effectiveness of interventions is accounting for the personal variation in relation to theoretical ideals. Specifically, increased SDM may be assumed to be the ideal approach; however, many patients may not wish to be proactively involved and may wish to delegate decisions to clinicians.^59^ Similarly, a low level of decisional conflict may not lead directly to satisfaction and quality decision making. In addition, capturing a person's values and preferences through SDM may not lead to a reduction in decisional conflict or an increase in satisfaction. A range of outcome measures in combination may help account for this personal variation.

### 4.3 Using SDM for patients or surrogate decision makers

Though SDM focuses on patients, a similar process could also be used with family surrogate decision maker where they can legally fulfil this role. The focus here should be on establishing and implementing an approach which aligns with the PLwD’s values and preferences. Where patient/carer dyads are involved in the studies reviewed, the patient and carer's roles are often conflated to provide a single measure of involvement, despite there being two people involved with different roles. This limits the understanding of how the PLwD is specifically supported by a family member and how much autonomy is retained by the PLwD. The focus on surrogate decision makers seems to be based on the blanket assumption that the PLwD cannot make their own decisions. Though some PLwD will lack this ability, this is infrequently discussed and there is little evidence that any specific processes or mechanisms can effectively support the PLwD to participate to a greater extent in decisions about their care.

### 4.4 Advance Care Planning and the ‘here and now’

In evaluating ACP interventions longer‐term follow‐up becomes more methodologically challenging. ACPs relate to anticipated events yet the anticipated scenario expressed in the ACP may differ to the reality being faced by a substitute or surrogate decision maker. Here, a decision maker may need to deviate somewhat from the wishes expressed in the ACP.^22^ The anticipated but variable progression of dementia over time may explain why the intervention detailed by Hanson *et al,* ^40^ only led to greater concordance between decision makers and health‐care providers at a nine‐month follow‐up and why no change in care‐related agreement arose in the study presented by Whitlatch *et al* ^44^ Family decision makers at an earlier stage may have more optimistic expectations of their relative's cognitive decline than medical professionals. With time, the expectations of the family member may converge with those of professionals hence why alignment in perspectives occurs only when significant time has passed.

### 4.5 Decision Making by Clinicians and the Absence of Family Support

The focus on family decision makers detracts from the role of clinicians who may need to make decisions on behalf of patients. Specific interventions could support clinicians to obtain information on a person's values as part of either SDM or to support best interests discussions. Only the ‘WeDECide’ interventions^39^, ^43^ focus on health‐care professionals by training them to engage in SDM. Though this training proved beneficial, it relates specifically to ACP not to here‐and‐now decisions. In some contexts, clinicians involved in decisions may face conflict and uncertainty in deciding what may be in a PLwD’s best interests.^60^, ^61^ A further issue arises where no appropriate family surrogate decision maker can be identified or where no insight into the person's values or preferences can be gained. In these situations, a time‐specific evaluation of the person's best interests is made, and independent assessment and support can prove valuable.^62^ No interventions were identified to support decision making where the views and preferences of a PLwD cannot be established.

### 4.6 The role of study context

Reflecting on the studies included it becomes clear that many are highly specific to the context in which they were devised and tested. This may support implementation but can also limit the transferability of interventions to new settings. If any of the interventions were to be modified and implemented into a new context, their effectiveness would need to be demonstrated in this new setting. Ideally, effectiveness would be determined by measuring of decision process and decision quality related to the PLwD, surrogate or both separately based on the PLwD’s decisional capacity. If new interventions are designed for specific decisions, settings or individuals, the involvement of PLwD, carers and health‐care professionals will be crucial in ensuring any interventions produced are acceptable to all those they aim to support.^63^

### 4.7 Strengths and limitations

The search criteria were intentionally broad but specifically limited to dementia. A broader search using terms such as ‘older adults’ may have identified additional studies yet produced an unmanageable number of results. Full‐text reviews were primarily undertaken by one author only; however, this was mitigated by in‐depth discussions about suitability of inclusion with an additional author. The search found six studies focusing on ACP; though these are valuable, ACP was not the primary intended focus of the review. No studies demonstrated effectiveness in supporting decisions beyond tube feeding that may arise in the here and now for PLwD.

## 5 CONCLUSION

Certain interventions can improve various aspects of the decision‐making process with, or for, PLwD. Establishing goals of care formally can improve concordance of goals between family members and clinicians over time, hence improving the quality of a decision made. The interventions focus primarily on a substitute decision makers as opposed to a PLwD and no approaches were effective in improving decisions made by PLwD themselves or making decisions align with their values. There is a lack of evidence demonstrating that preference elicitation at earlier stages of dementia positively impacts the quality of any real‐world decisions made at a later stage either with or for a person living with dementia. Interventions need to be designed and evaluated that support the PLwD in contributing to decisions throughout the progression of dementia.

## AUTHOR CONTRIBUTIONS

AGR led the project, drafted the protocol and revised it following input from the remaining authors, acted as primary reviewer for all articles, and drafted the manuscript. LE designed the search strategy and acted as second reviewer screening papers by title and abstract. RT supported the design of the review and methods, and supported decisions of study inclusion where AGR and LE were uncertain. CE, RRW and CA supported the protocol development and provided oversight on the wider study. All authors commented on drafts of the manuscript, supported the response to peer reviewer's comments and approved the final manuscript.

## Data Availability

Data sharing is not applicable to this article as no new data were created or analysed in this study.

## APPENDIX 1 Search Strategy used in Medline via OVID

### Search Strategy used in Medline via OVID

1. Exp Dementia/
2. Dement*.mp
3. Alzheimer*.mp
4. Lewy bod*.mp
5. Exp Neurocognitive disorders/
6. 1 or 2 or 3 or 4 or 5
7. Decision Making/
8. Decision*.mp
9. Informed consent/
10. 7 or 8 or 9
11. 6 and 10
12. Limit 11 to (English language and humans)
